# Effects of glutathione on the physicochemical properties of high hydrostatically pressure gelatinized maize starch

**DOI:** 10.1016/j.fochx.2025.102158

**Published:** 2025-01-03

**Authors:** Wei Zhang, Danxia Shi, Wenming Dong, Hong Li, Xiaohui Liu

**Affiliations:** a College of Food Science and Technology, Yunnan Agricultural University, Kunming 650500, China; b College of Tea Science, Yunnan Agricultural University, Kunming 650500, China

**Keywords:** Maize starch, GSH, HHP, Chain distribution, Gelatinization

## Abstract

This research prepared gelatinized waxy maize starch (WMS), low-amylose maize starch (LAS), and high-amylose maize starch (HAS) with different glutathione (GSH) content (5, 10, and 15 %) using high hydrostatic pressure (HHP) at 600 MPa. Scanning electron microscopy (SEM) revealed damaged morphology of WMS and complete swelled granules of LAS and HAS with different degree of gelatinization (DG) values, 92.86, 59.36, and 17.45 %, respectively. Fourier transform infrared spectroscopy (IR spectra), laser confocal micro-Raman (LCM-Raman) spectroscopy, and X-ray diffraction (XRD) results suggested that the crystallinity content of gelatinized WMS and HAS with addition of GSH was higher than that of LAS, and the gelatinized LAS and HAS were mainly of C type and V type, respectively. The resistant starch of LAS (25.15 %) and HAS (34.76 %) increased with GSH addition. The crosslinking between GSH and amylose/amylopectin caused changes in physicochemical properties. This study will provided theoretical basis for GSH usage in food industry.

## Highlights

• Physicochemical properties of maize starch co-gelatinized with GSH by HHP were changed.
• The DG of starch was primary related to amylose/amylopectin ratios.
• The crosslinking between GSH and amylopectin increased the contents of RS.

## 1 Introduction

Maize (*Zea mays* L.) is an important cereal crop with abundant production, extensively used in human diets and food industrial materials, especially in cooked and baked products (Holmes et al., 2019). However, the native starch is limited in industry due to the its poor solubility, mechanical shearing resistance, susceptibility to retrogradation, and crystallization (Xu et al., 2024). In order to overcome deficiencies, hydrocolloids (Li, Xie, et al., 2019), amino acids (Gałkowska & Juszczak, 2019), salts (Wang et al., 2017) and lipid (Oyeyinka et al., 2021) are added to improve the characteristics of the starch. Due to demands in food processing, the maize strach usually undergoes gelatinization, the recrystallization of starch chains with hydrogen bonding or the double-helical crystallites usually lead to resistant starch (RS). In contarst to appearance transformation, the gelatinization process of starch and nutritients in food system need further investigation to develop with desirable properties.

Starch gelatinization is typically achieved by heating with excess water, resulting in granular melting, swelling of crystallites, and a progressive increase in viscosity (Yu et al., 2016). In addition to thermal gelatinization**,** high hydrostatic pressure (HHP) is a promising technique in the food industry for physical modification of starch. HHP is reported to break or change non-covalent linkages with the starch granules with no impact on covalent interactions, without any specific temperature, the proper pressure, the HHP treatment could change A-type starches into B-type starches and hydrate the crystalline regions and amorphous region, which are formed by short range periodic order in helical conformation and helices branches, respectively (da Costa Pinto et al., 2021; Zhi-Guang et al., 2020).

Previous studies have indicated that the degree of gelatinization (DG) mainly depends on the applied pressure, temperature, time, and the botanical sources of starch. An important factor influencing DG is the structural character of starch. At the micron-sized scale, starch granules are described as complexes of crystalline lamellae, semi-crystalline growth rings and amorphous lamellae. The crystalline lamellae are divided into A type, B type, and C type based on the arrangement of amylopectin double-helical structures (Rodriguez-Garcia et al., 2021). At the supramolecular scale, the amorphous and crystalline regions are respectively constituted by amylose and amylopectin, contributing to granule morphology, lamellar, and fractal structure (Zhang et al., 2014). The amylose/amylopectin ratios affect the size, and crystalline structure, thus resulting in various gelatinization properties during thermal gelatinization (Park et al., 2023). Of particular interest is the regulation of the gelatinization process through the addition of other components such as polyphenols, proteins, lipids, and pectin, which is common in food industry processing (Abdel-Aal et al., 2020; Amoako & Awika, 2019; Lian et al., 2014; Ma et al., 2019).

As a legal food additive in China, glutathione (GSH) is an effective endogenous antioxidant for mitigating browning and the flavor loss. It also has positive effects on human diseases such as cardiovascular and inflammatory diseases (Webber et al., 2017; Yao et al., 2021; Zhang et al., 2013). The interaction between starch and protein has been extensively studied to improve the properties of the starch-protein system, primarily involving electrostatic complexing and hydrogen bonding (Zhan et al., 2020). Starch-protein conjunctions lead to increased ordered structure and retardation of starch digestion (Li et al., 2024). Despite the wide-ranging studies on starch-protein interactions, as a small molecule peptide, GSH has been long term ignored, with no prior studies concerning the starch-GSH mixture in HHP-induced gelatinization. The objectives of this study were to investigate the difference in properties of gelatinized maize starch and GSH with different amylopectin contents and the molecular interaction between starch and GSH, the results of this study will provide the theoretical understanding necessary for mixture of starch and GSH in potential industrial applications.

## 2 Materials and methods

### 2.1 Materials

Three types of MS with different amylose content (AC)—waxy maize starch (WMS), low-amylose maize starch (LAS), and high-amylose maize starch (HAS) were purchased from Beijing Xiangyun Biochemical Co., Ltd. (Beijing, China). The moisture contents of WMS, LAS and HAS were approximately 11.60, 12.81, and 13.50 %, respectively. Their ACs 7, 56, and 71 %, respectively, based on data provided by Beijing Xiangyun Biochemical Co., Ltd. GSH with high performance liquid chromatography (HPLC) purity ≥98.0 %, Dimethyl sulfoxide d_6_ (DMSO *d*_6_ with GC purity ≥99.0 %, L-(−)- Glucose, and α-Amylase from porcine pancreas (700–1400 U/mg protein) were provided by Sigma Aldrich (St. Louis, USA). Rice amylose standards were provided by China National Rice Research Institute (Hangzhou, Zhejiang, China), while the oligo-saccharides kit was obtained from Elicityl (Grenoble, France). All other chemical reagents were of analytical grade and were purchased from Sinopharm Chemical Reagent Co., Ltd. (Shanghai, China).

### 2.2 Preparation of gelatinized starch

The gelatinization of MS induced by HHP was performed following the method introduced by Li, Yuan, et al. (2019) with some modifications. Briefly, 15 g of WMS, LAMS, and HAMS were suspended in 150 mL of distilled water to achieve a water content of 10 % (*w*/*v*) in polypropylene bags, respectively. GSH at different ratios (5, 10, and 15 % *w*/w, based on starch) was then added to the suspensions. The polypropylene bags were vacuum-sealed and thoroughly mixed before subjected to the HHP equipment under a pressure level of 600 MPa for 20 min at 25 °C (HHP L1–600/20, Huatai Senmiao UHV Equipment Engineering Technology Co., Ltd., Tianjin, China). Pressurization and decompression rates were set at 8 MPa/s. The resulting gelatinized starch samples were freeze-dried, ground into powder, screened through a 100-mesh sieve and stored in sealed containers at 4 °C for further analysis. Based on the added levels of GSH ranging from 0 to 15 %, the gelatinized starch samples were marked as WMS-GSH-0 to 15, MAS-GSH-0 to 15, HAS-GSH-0 to 15, respectively. Gelatinized starch samples without GSH served as control groups.

### 2.3 Physicochemical properties

#### 2.3.1 Particle size and morphology

The average volume-weighted mean diameter (D_4,3_) of the samples was measured by using a laser particle size distribution analyzer (Microtrac S3500, Micotrac Inc., USA).

The morphology of the starch was examined using a scanning electron microscopy (SEM) (Nova Nano 450, FEI, USA) at an accelerating voltage of 10 kV. Prior to observation, starch samples were fixed onto a double-sided adhesive tape adhered to a circular metal subbase and coated with gold under vacuum using an ion sputter coater (JFC-1600, Hitachi, Japan) for 30 s.

#### 2.3.2 Molecular distribution

The weight average molecular weight (M_w_) of the starch was determined using high-performance size-exclusion chromatography (HPSEC) separation (Agilent 1260, CA, USA) equipped with a multi-angle laser-light scatering detector (DAWN HELEOS II, Wyatt Technology, CA, USA) and a refractive-index detector (Optilab T-rEX, Wyatt Technology, CA, USA). The column series used for HPSEC separation were Ohpak SB-803 HQ, Ohpak SB-805 HQ, and 804 HQ (Shodex, Showa Denko K.K., Tokyo, Japan). Starch samples were prepared for HPSEC analysis according to Ma et al. (2021). A 100 μL sample was injected at 0.4 mL/min using 0.1 M NaNO_3_ as the mobile phase and a column oven temperature of 60 °C. Data analysis was performed using ASTRA software (version 5.3.1.5, Wyatt Technology, Santa Barbara, USA) based on the Mark-Houwink Equation.

#### 2.3.3 Degree of branching (DB)

The DB of starch samples was determined using a nuclear magnetic resonance (NMR) spectrometer (Biospin GmbH, Bruker, Rheinstetten, Germany) in 5 mm NMR tubes (Bai et al., 2011). The ^1^H NMR spectra were acquired at 500.23 MHz with 32 scans. Starch samples (10 mg) were dissolved in DMSO *d*_6_ (1 mL) and heated at 80 °C for 24 h, followed by centrifugation at 12,000 rpm for 10 min. The supernatant was then prepared for testing. The date were analyzed using MestReNova software (Mestrelab Research, Santiago, Spain) and calculated according to the formulation:

$$\mathrm{DB}\left(\%\right)=\frac{A_{I-1,6}}{A_{I-1,6}+A_{I-1,4}}*100$$

where the A_I-1,6_ and A_I-1,4_ represent the area of α-1,6 and α-1,4 glycosidic bond.

#### 2.3.4 ACs

The AC of starch samples was determined by using enzyme-linked immunosorbent assay (ELISA) (Multiskan GO, ThermoFisher, USA) at 720 nm resulting from the reaction of starch with iodine (Jiang et al., 2015). Rice amylose was used as the standard.

#### 2.3.5 Chain length distribution

The number-average degrees of polymerization (${\bar{\mathrm{DP}}}_{n}$) of the starch samples were determined using gel permeation chromatography (GPC, ICS500+, Thermo Fisher Scientific, USA) equipped with a CarboPac PA10 column, following a previous report method with modifications (Sorndech et al., 2016). The samples (5 mg) were suspended in distilled water (5 mL) and boiled for 60 min with continuous stirring. After cooling to 50 °C, sodium acetate (NaAc, 125 μL), sodium azide (NaN_3_, 5 μL), and isoamylase (5 μL) were added to the paste. The mixture was then incubated at 38 °C for 24 h and boiled for 20 min to inactivate the isoamylase. The debranched starch (600 μL) was dried under nitrogen at 25 °C for further analysis.

The mobile phase consisted of 200 mM sodium hydroxide (NaOH) for phase A and a mixture of 200 mM NaOH and 200 mM NaAc for phase B, with a flow rate of 0.3 mL/min at 30 °C. A linear gradient was applied as follows: the system was held at 90 % A for 2 min, followed by a decrease from 90 % to 40 % A over 12 min. Subsequently, it was held at 40 % A for 3 min before shifting back to 90 % A immediately, where it was maintained for 5 min. The dried starch was dissolved in 600 μL of phase B solution and centrifuged at 10,000 ×*g* for 10 min. The supernatant (20 μL) were used for analysis.

### 2.4 Fourier transform infrared spectroscopy (IR spectra)

The IR spectra of the samples were acquired using a IR spectrometer (Nicolet 6700, Thermo Fisher, USA). Starch samples were mixed with potassium bromide (KBr, 1 %, *w*/w) and ground to a powder, then pressed into transparent pellets. Each spectrum was recorded between 4000 and 400 cm^−1^ at a resolution of 4 cm^−1^, with 16 scans averaged for each measurement.

### 2.5 Laser confocal micro-Raman (LCM-Raman) spectroscopy

The starch samples were analyzed using a Raman microscope system (Renishaw InVia, Renishaw, UK) equipped with a Leica microscope (Leica Biosystems, Wetzlar, Germany), and a 785 nm green diode laser source was used. Spectra were obtained from at least six different positions of each sample in the range of 3200–100 cm^−1^ with a resolution of approximately 7 cm^−1^. The short-range ordered structure in starch was assessed by calculating the full width at half-maximum (FWHM) of the band at 480 cm^−1^ using the WIRE 2.0 software (Wang, Li, et al., 2015).

### 2.6 X-ray diffraction (XRD)

The crystalline structure of the starch samples was analyzed using XRD with a D8 Advance instrument (Bruker, Germany), following the method by Liu et al. (2020). The XRD was operated at 40 kV and 40 mA to collect data over the range of 5° to 40° (2θ) at a scanning rate of 2°/min and a step size of 0.02°.

### 2.7 Thermal properties

Differential scanning calorimetry (DSC) tests were performed using a differential scanning calorimeter (Discovery DSC 2500, TA, USA) equipped with a thermal analysis station (Wang et al., 2014). Starch samples (2 mg) were accurately weighted into aluminum sample pans. Distilled water was added to achieve a water-to-starch ratio of 3:1 (*w*/w). The pans were sealed and equilibrated overnight at room temperature. The samples were then heated from 20 to 120 °C at a rate of 10 °C/min with an empty pan serving as used as the reference. Thermal transition parameters including onset temperature (*T*_0_), peak temperature (*T*_p_), conclusion temperature (*T*_c_), and enthalpy change (Δ*H*) were obtained using data recording software. The DG was calculated as:

$$\mathrm{DG}\;\left(\%\right)=\left[\left({\text{∆H}}_{0}–{\text{∆H}}_{1}\right)/{\text{∆H}}_{0}\right]\times 100$$

where ΔH_0_ is the gelatinization enthalpy of native starch (J/g) and ΔH_1_ is the gelatinization enthalpy (J/g). DG values of 100 % indicates completely gelatinized starch.

### 2.8 Pasting viscosity

The viscosity properties were assessed using a rapid visco analyzer (RVA4500, Perten Instrument, Stockholm, Poland). Starch (2.50 g) and lipids (125 mg) were sequentially weighed into the RVA canisters, and distilled water was added to reach a total weight of 28 g. The mixtures were initially stirred with the plastic paddle and then in the instrument at 960 rpm for the first 10 s, followed by continuous stirring at 160 rpm until the completion of 13-min STD1 profile provided with the RVA. The sample was held at 50 °C for 1 min, then heated from 50 to 95 °C at a rate of 12 °C /min, head at 95 °C for 2 min, followed by cooling to 50 °C at the same rate, and held at 50 °C for 7 min. The peak viscosity (PV), trough viscosity (TV), breakdown (BD) viscosity, final viscosity (FV), and setback (SB) viscosity were recorded.

### 2.9 Resistant starch (RS) content

The RS content was determined following the method previously described by Yang et al. (2006). It was calculated as the difference between the total starch and the starch hydrolyzed by enzymes. In brief, a starch sample (4 mg) was digested with 4 mL of α-amylase for 16 h at 37 °C. The residues were then centrifuged (5000 ×*g*, 10 min) and washed with ethanol before hydrolyzed with 2 mL of potassium hydroxide (KOH) for 20 min. Subsequently, 8 mL of sodium acetate solution (1.2 M) was added, and the sample was digested with amyloglucosidase for 30 min at 50 °C. The RS content was calculated based on the released glucose using a glucose oxidase/ perioxidase (GOPOD) kit.

### 2.10 Statistical analysis

All experiments were performed at least in triplicate and the results are presented as mean ± standard deviation. For XRD analysis, a single measurement was conducted. Analysis of variance (ANOVA) following Duncan's test (*p* < 0.05) was conducted using the SPSS 10.0 Statistical Software Program (SPSS Inc., Chicago, IL, USA).

## 3 Results and discussion

### 3.1 Morphology and particle size

**Fig. 1:**
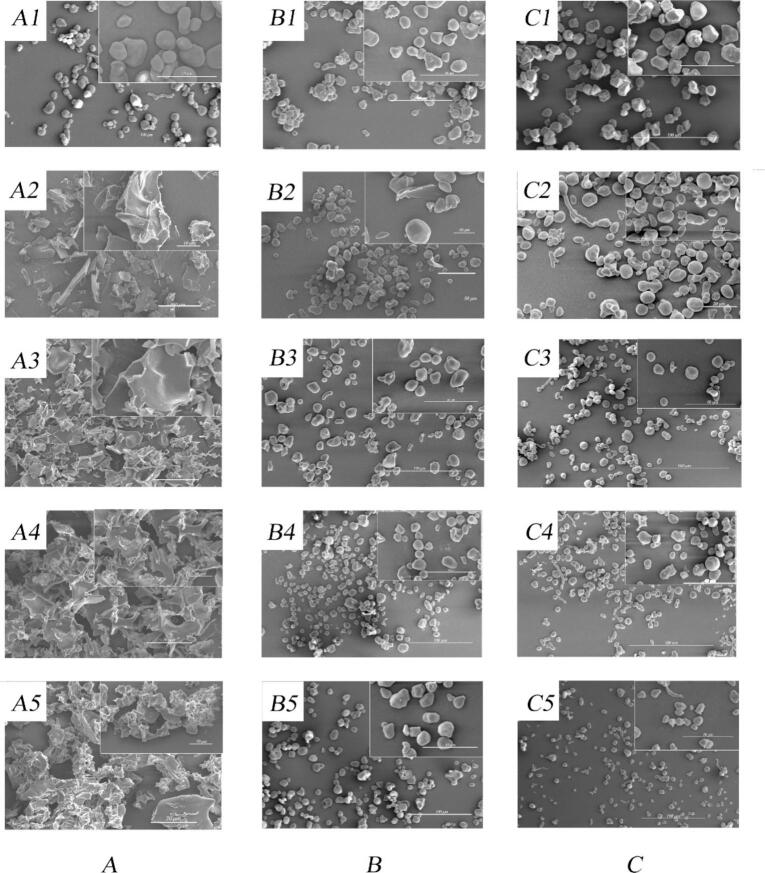
SEM images of native and HHP gelatinized starch. A1 to A5: native WMS, WMS-GSH-0 to WMS-GSH-15; B1 to B5: native LAS, LAS-GSH-0 to LAS-GSH-15; C1 to C5: native HAS, HAS-GSH-0 to HAS-GSH-15.

The morphology of native and gelatinized starch was shown in Table 1 and Fig. 1. The WMS starch samples exhibited completely disrupted granule morphology, whereas LAS and HAS samples retained identifiable granule shapes with various degrees of distortion, indicating starch gelatinization under HHP treatments, especially evident in WMS. Previous studies have shown that starch gelatinized by HHP retains a better granular structure compared to heat-induced gelatinization, with granular integrity closely related to AC (Li & Zhu, 2018; Yang et al., 2017). The degree of starch damage decreased with increasing AC, consistent with existing literature (Jeong et al., 2021; Li & Hu, 2021).

**Table 1:** Structural characteristics and particle size of starch samples gelatinized by HHP.

Sample	D_4,3_ (μm)	Mw (×10^8^ g/mol)	Polydispersity	DB (%)	Amylose content (%)
WMS					
Native	16.42 ± 0.27^e^	8.03^a^	1.17 ± 0.17^d^	1.01	7.07 ± 0.13^a^
WMS-GSH-0	43.66 ± 0.21^a^	4.07^d^	1.13 ± 0.16^d^	4.81	6.87 ± 0.19^b^
WMS-GSH-5	37.68 ± 0.14^b^	3.28^e^	2.47 ± 0.09^c^	2.00	5.59 ± 0.13^c^
WMS-GSH-10	31.57 ± 0.12^c^	5.51^b^	3.84 ± 0.06^b^	4.53	4.74 ± 0.19^d^
WMS-GSH-15	26.23 ± 0.14^d^	5.14^c^	15.46 ± 0.12^a^	13.04	4.01 ± 0.13^e^

LAS					
Native	14.86 ± 0.01^e^	3.57^a^	7.50 ± 0.12^c^	0.98	53.58 ± 0.25^d^
LAS-GSH-0	18.1 ± 0.02^a^	2.87^c^	8.39 ± 0.11^b^	0.91	59.07 ± 0.06^a^
LAS-GSH-5	17.11 ± 0.01^b^	1.34^e^	6.44 ± 0.12^d^	2.05	58.00 ± 0.19^b^
LAS-GSH-10	15.56 ± 0.03^d^	3.28^ab^	8.56 ± 0.09^a^	2.31	58.20 ± 0.19^ab^
LAS-GSH-15	16.47 ± 0.17^c^	1.75^d^	7.67 ± 0.09^c^	1.13	54.86 ± 0.06^c^

HAS					
Native	11.92 ± 0.05a	2.32^b^	5.59 ± 0.11^b^	1.01	70.33 ± 0.19^c^
HAS-GSH-0	10.31 ± 0.10^d^	1.92^c^	5.49 ± 0.11^b^	0.35	76.25 ± 0.13^a^
HAS-GSH-5	10.64 ± 0.04^bc^	2.93^ab^	6.10 ± 0.10^a^	0.54	75.90 ± 0.18^a^
HAS-GSH-10	10.98 ± 0.08^b^	3.06^a^	6.02 ± 0.11^a^	0.61	72.92 ± 0.19^b^
HAS-GSH-15	10.36 ± 0.20^d^	1.96^c^	6.04 ± 0.11^a^	1.45	71.21 ± 0.13^c^

Values are expressed as means ± standard deviations expected for DB. Different letters represent a significant difference between data in the same amylose/amylopectin ratio (*p* < 0.05).

In gelatinized WMS, granules changed into separated sheet structures without GSH, while with increasing GSH, hair-like structures appeared at the edges of granules, along with smaller pieces, suggesting that GSH could interact with amylopectin, influencing the formation of starch network. In gelatinized LAS and HAS, the relative integrated granules suggest that GSH and water permeated into the particles. Notably, hair-like structures only appeared at GSH content up to 15 %, suggesting a preference for interaction with amylopectin as amylose disorder increases. Particle size of starch samples was closely related to AC and GSH content (Table 1). However, D_4,3_ of WMS decreased sharply with increasing GSH, consistent with granule morphology observed by SEM. Granules of LAS and HAS maintained particle morphology, with minimal variation in D_4,3_, suggesting GSH-starch interaction occurred within the particle. The swelling behavior of LAS was slightly restricted by GSH, indicating competitive permeation of granules with water.

### 3.2 Supramolecular structure

#### 3.2.1 Molecular character

The weight-average molar mass (M_w_), polydispersity (M_w_/M_n_) and DB were showed in Table 1. The M_w_ of MS with different amylopectin content decreased after HHP gelatinization, indicating disruption of crystallites and the rearrangement of starch, consistent with findings by Zeng et al. (2018). Addition of GSH led to a decrease in the M_w_ of WMS to 3.28 × 10^8^ g/mol, followed by an increase to 5.51 × 10^8^ g/mol at 10 % (*w*/w) GSH, suggesting GSH interaction with amylopectin and starch disintegration. Both LAS and HAS reached peak Mw as GSH increased to 10 %. However, the Mw of HAS had a sharp decrease as the content of GSH reach to 15 %, indicating potential hydrogen bond formation with amylose, affecting the rearrangement of starch and aggravating degradation. Polydispersity index (PDI) is defined as the ratio of weight-average molar mass to the number-average molar mass and an indicator of M_w_ distribution (Zhang et al., 2014). The highest value of WMS-GSH-15 confirmed that the rearrangement of WMS was intervened by GSH, forming more low-DP molecular chains (Zeng et al., 2015). The PDI values of LAS initially increased then decreased with increasing GSH, while HAS PDI remained constant. The results suggested GSH-induced cross-linking between amylose/amylopectin and GSH were affected by the content of GSH.

To further characterize the structure of starch, ^1^H NMR spectroscopy determined the DB, as the chemical shift of anomeric protons in α-(1,4)-glycosidic bonds differs from that of α-(1,6)-glycosidic bonds (Hernandez et al., 2008). Table 1 shows an increased DB for all HHP-gelatinized starch compared to native starch, particularly WMS-GSH-15, suggesting α-(1,4)-glycosidic bond degradation initially, with GSH playing a role in preserving α-(1,6)-glycosidic bonds. However, α-(1,6)-glycosidic bonds in amylopectin chains degraded first with increasing AC at a low level of GSH.

AC is an important structural factor influencing functions, such as RS and retrogradation rate, owing to its high mobility and low branching hindrance (Li, Yuan, et al., 2019; Wang, Wang, et al., 2015). Table 1 indicates that the AC of WMS was 7.07 %, and HHP-induced gelatinization significantly decreased AC with increasing GSH. Similar trends were observed in HAS samples, suggesting GSH affected the rearrangement by interacting with amylopectin. However, the cross-linking process is influenced by the supramolecular interaction between amylose and amylopectin, as reflected in LAS-GSH-0, LAS-GSH-5, and LAS-GSH-10, where AC significantly decreased with the addition of GSH up to 15 %.

#### 3.2.2 Amylopectin chain length distribution

**Fig. 2:**
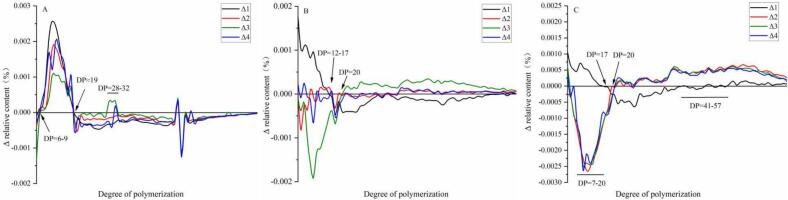
DP of native and HHP gelatinized starch with GSH. A: WMS, Δ1-Δ4 represent WMS-GSH-0 to WMS-GSH-15; B: LAS, Δ1-Δ4 represent LAS-GSH-0 to LAS- GSH-15; C: HAS, Δ1-Δ4 represent HAS-GSH-0 to HAS-GSH-15.

To analyze GSH's role in the rearrangement of amylopectin, four regions of amylopectin were distinguished: A chains (6 < DP ≤ 12), B_1_ chains (13 < DP ≤ 24), B_2_ chains (25 < DP ≤ 36), and B_3_ chains (37 < DP ≤ 100) (Wang et al., 2022). The amylopectin chain-length distribution of maize starch is shown in Table 2 and the chromatography profile is shown in Fig. 2.Higher relative amounts of B_1_ chains were clearly observed in all starch samples, indicating predominant B-type crystallization, favored by a minimum DP of 10 and preferably 13 for a double helix (Gidley & Bulpin, 1987). As amylopectin decreased, A chains decreased while B_2_ and B_3_ chains increased. The addition of GSH at low levels (<10 %) significantly increased the amount of B_2_ and B_3_ chain amounts in gelatinized WMS. Although the amylopectin chain-length distribution of LAS was slightly affected by the addition of GSH, the amount of A chains in HAS significantly decreased and that of the B_3_ chains increased slightly. These findings suggest that GSH combined the short chains resulting in the aggregation of amylopectin helices, enhancing resistance to digestion (Chang et al., 2021).

**Table 2:** Amylopectin chain length distribution of maize starch gelatinized by HHP.

Sample	Amylopectin chain length distribution (%)				${\bar{\mathrm{DP}}}_{n}$
	6<DP ≤ 12	13<DP ≤ 24	25<DP ≤ 36	37<DP ≤ 100	
WMS					
Native	23.48 ± 0.07^d^	50.47 ± 0.31^c^	14.41 ± 0.12^a^	11.64 ± 0.13^i^	21.00 ± 0.14^a^
WMS-GSH-0	24.33 ± 0.20^a^	50.84 ± 0.50^ab^	14.02 ± 0.07^c^	10.81 ± 0.03^l^	20.59 ± 0.12^cd^
WMS-GSH-5	23.94 ± 0.03^c^	50.86 ± 0.20^a^	14.24 ± 0.11^ab^	10.96 ± 0.24^k^	20.69 ± 0.02^c^
WMS-GSH-10	23.62 ± 0.17^cd^	50.78 ± 0.13^b^	14.44 ± 0.01^a^	11.16 ± 0.32^j^	20.81 ± 0.02^b^
WMS-GSH-15	24.08 ± 0.08^b^	50.84 ± 0.32^a^	14.13 ± 0.33^bc^	10.95 ± 0.02^k^	20.66 ± 0.17^c^

LAS					
Native	14.82 ± 0.02^b^	46.96 ± 0.11^a^	17.84 ± 0.32^bc^	20.66 ± 0.05^bc^	25.51 ± 0.01^b^
LAS-GSH-0	15.54 ± 0.15^a^	46.48 ± 0.13^b^	17.68 ± 0.67c	20.30 ± 0.06^c^	25.29 ± 0.03^c^
LAS-GSH-5	14.66 ± 0.14^c^	46.68 ± 0.10^a^	17.80 ± 0.06^b^	20.86 ± 0.02^b^	25.59 ± 0.02^b^
LAS-GSH-10	14.16 ± 0.12^d^	46.24 ± 0.20^b^	18.09 ± 0.06^a^	21.51 ± 0.11^a^	25.91 ± 0.24^a^
LAS-GSH-15	14.77 ± 0.11^b^	46.53 ± 0.02^a^	17.83 ± 0.02^bc^	20.87 ± 0.12^b^	25.59 ± 0.34^b^

HAS					
Native	9.37 ± 0.12^b^	39.09 ± 0.03^i^	21.43 ± 0.05^a^	30.11 ± 0.04^d^	29.89 ± 0.03^c^
HAS-GSH-0	9.82 ± 0.18^a^	38.97 ± 0.25^j^	21.01 ± 0.13^b^	30.20 ± 0.02^d^	29.84 ± 0.05^c^
HAS-GSH-5	8.41 ± 0.01^c^	37.94 ± 0.05^l^	21.60 ± 0.04^a^	32.05 ± 0.03^a^	30.77 ± 0.15^a^
HAS-GSH-10	8.54 ± 0.22^c^	37.94 ± 0.22^l^	21.62 ± 0.10^a^	31.90 ± 0.04^b^	30.69 ± 0.20^b^
HAS-GSH-15	8.63 ± 0.02^c^	38.03 ± 0.18^k^	21.60 ± 0.27^a^	31.74 ± 0.15^bc^	30.61 ± 0.06^b^

Values are expressed as means ± standard deviations. Different letters represent a significant difference between data in the same amylose/amylopectin ratio (*p* < 0.05).

### 3.3 Crystalline properties

#### 3.3.1 Short-range molecular order

**Fig. 3:**
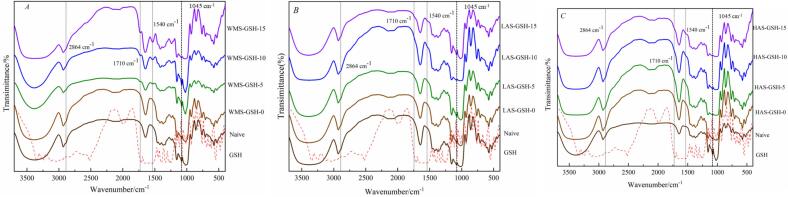
Fourier transform infrared spectra of native starch and gelatinized starch samples: A represented WMS samples; B represented LAS samples; C represented HAS samples.

IR spectra and LCM-Raman were used to assess the short-range molecular order of starch samples (Wang, Zhang, et al., 2016). The IR spectrum of native and gelatinized WMS, LAS, and HAS is shown in Fig. 3. The stretching vibrations of free hydroxyl (3500–3700 cm^−1^) and bonded hydroxyl groups (3200–3500 cm^−1^) were responsible for absorbance at 3200–3700 cm^−1^ (Wang et al., 2020). In gelatinized samples, particularly LAS and HAS, both groups became wider compared to native starch. The results indicated that GSH contains numerous hydroxy groups facilitating intermolecular and intramolecular crosslinking, such as the double-helix structure in amylopectin during gelatinization. The absorbance at 2864 cm^−1^, assigned to CH-stretching vibration, showed no difference between native and gelatinized samples, indicating no formation of chemical bonds (Bai et al., 2009). The absorbance at 1710 cm^−1^ indicated a cross-linking reaction of GSH and starch, especially evident in WMS with a high GSH level. The increasing FWHM values of Raman band at 480 cm^−1^ suggested progressively increasing short-range molecular order upon gelatinization by HHP without GSH (Liu et al., 2021). As GSH increased, a slightly decreasing degree of short-range molecular order was observed, which suggested GSH can promote the increase of double helix structure in WAS and inhibit the recrystallization of starch. However, the degree of short-range molecular order for LAS and HAS remained independent of gelatinization by HHP and GSH, indicated with the low content of amylopectin, the GSH could not reduce the recrystallization.

#### 3.3.2 XRD pattern and RC

**Fig. 4:**
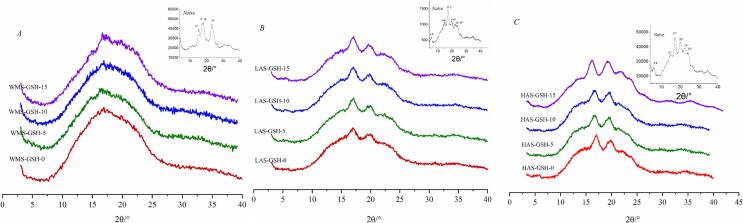
XRD patterns of WMS (A), LAS (B), HAS (C) with different contents of GSH.

Linea and branched chains usually organize into helix conformation and subsequently formed into bravais lattice and unique crystalline structures (Krishnan et al., 2021). XRD analysis was used to investigate the crystallization and long-range ordering of starch samples gelatinized with GSH by HHP (Fig. 4). The diffraction patterns of native WMS and starch pregelatinized with GSH did not significantly differ, and with characterizatic single peaks at around of 15° and 23° (2*θ*) and double peaks at around 17° and 18°, which being identfied as type A. While native MAS and HAS showed a B-type XRD pattern with obvious diffraction peaks at 5°, 6°, 15°, 17°, 20°, and 22° (2*θ*) as shown in Fig. 4. There were no sharp peaks in all the gelatinized WMS with degrees of crystallinity ranging from 12.46 % to 14.57 % (Table 3), indicating primarily amorphous states and reduced relative crystallinity (RC) values. This was consistent with the results of sorghum starch, suggesting decreased crystallinity attributed to disruption of double helices in amylopectin crystallites caused by water penetration and the GSH could slightly increase the RC values which indicated the interaction between GSH and amylopectin (Liu et al., 2016). As for LAS, there were obvious peaks at 15° with an indistinguishable double peak at 17,18° and a small peak at 23°, indicating a weakened A-type starch, which was reported to with orthorhombic crystal structure (Rojas-Molina et al., 2024). The results suggested that the single structure of WMS, the interact with GSH has no impact on the formation of A type starch. These type changes might result from the degradation of the crystalline region and crystallite orientation shifts influenced by GSH. RC values of all LAS samples decreased after gelatinization, with lower values at higher GSH levels (≥10 %), indicating increased amorphous region formation in the LAS/GSH complex. HAS had obvious peaks at 5.6^o^，15^o^，17^o^ and weak singlets at 22^o^ and 24^o^, which indicated a typical B-type starch. Additionally, V-type crystals，which is traditional defined as a versatile carrier for starch-lipid complexes with linear fatty acids for its single-helix crystalline structure, were present within the overall structure, which showed strong peaks at 7.1°, 12.2°, and 19.8°, This might be attributed to the aggregation of amylose and GSH with single-helix structure (Yu et al., 2016). It suggests the native HAS crystal structure shifted from typical B-type to multiple crystal, likely due to the shrinkage rearrangement of amylose and amylopectin affected by high pressure and GSH interaction. RC values of HAS sharply increased with increasing GSH, with lower values observed for WMS and LAS, indicating the significant role of helix formation between amylose and GSH (Qiao & Peng, 2024).

**Table 3:** Structural information of WMS, LAS and HAS.

Sample	FWHM 480 cm^−1^	RC⁎ (%)	DG (%)	Resistant starch (%)
WMS				
Native	15.11 ± 0.10^c^	20.24	/	0.26 ± 0.01#
WMS-GSH-0	21.13 ± 0.92^b^	12.46	96.54	0.26 ± 0.05#
WMS-GSH-5	19.96 ± 0.03^a^	13.39	91.02	0.25 ± 0.11#
WMS-GSH-10	19.85 ± 0.19^a^	14.29	92.86	0.27 ± 0.13#
WMS-GSH-15	17.21 ± 0.21^b^	14.57	92.56	0.24 ± 0.02#

LAS				
Native	15.43 ± 0.01#	23.15	/	37.68 ± 0.07^a^
LAS-GSH-0	15.79 ± 0.01#	15.94	24.79	19.53 ± 0.07^d^
LAS-GSH-5	15.67 ± 0.03#	15.97	26.65	23.76 ± 0.01^c^
LAS-GSH-10	15.53 ± 0.10#	13.94	59.36	24.97 ± 0.01^b^
LAS-GSH-15	15.77 ± 0.19#	13.40	35.48	25.15 ± 0.02^b^

HAS				
Native	15.20 ± 0.19^c^	22.10	/	43.87 ± 0.01^a^
HAS-GSH-0	15.91 ± 0.21^a^	12.88	6.94	33.13 ± 0.12^d^
HAS-GSH-5	15.51 ± 0.01^b^	16.86	7.22	34.76 ± 0.01^bc^
HAS-GSH-10	15.50 ± 0.02^b^	20.19	16.25	33.09 ± 0.22^d^
HAS-GSH-15	15.45 ± 0.04^b^	23.01	17.45	34.36 ± 0.12^c^

Values are means ± standard deviations except for the relative crystallinity.

⁎ : Relative crystallinity degree.

# : No significant difference.

### 3.4 Rapid viscosity

**Fig. 5:**
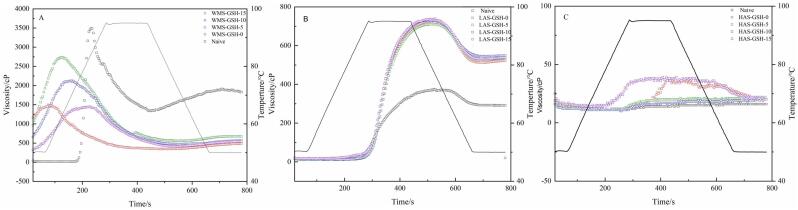
Pasting properties of HHP- gelatinized maize starch (WMS:A, LAS:B, HAS:C) at different GSH concentrations.

Pasting properties reflect the interaction between starch molecules and water during the temperature program. The effects of GSH concentrations on the pasting properties of starch are summarized inFig. 5 and the pasting profiles were presented in Table 4. As shown in Fig. 5, GSH content results an obvious reduction in pasting viscosity for WMS, including peak viscosity (PV), trough viscosity (TV), breakdown viscosity (BDV), final viscosity (FV), and setback viscosity (SBV). Furthe more, as the increase of GSH the pasting viscosity gradually increased. PV reflected the water-holding capacity of starch under shearing and swelling performance of granules (Li et al., 2022). The results of WMS indicated that starch treated with GSH had a weaker water-holding ability than native starch. The increase in PV under different GSH contents might be closely related to the changes induced by HHP. On the one hand, HHP treated WMS increased the short-range molecular order, resulting in a stronger resistance and water -holding ability under shearing and heating. One the other hand, a high proportion of amylopectin increas the radius of gyration of the molecules, which might increase in viscosity of the gelatinized starch paste. The interlink between GSH and amylopectin which contributed to maintaining the viscosity of WMS-GSH-15 (Han & Hamaker, 2001).

**Table 4:** Pasting properties of HHP- gelatinized maize starch at different GSH concentrations.

Sample	Peak viscosity (cP)	Through viscosity (cP)	Breakdown (cP)	Final viscosity (cP)	Setback (%)
WMS					
Native	3512 ± 21^a^	1351 ± 19^a^	2161 ± 9^a^	1827 ± 5^a^	476 ± 5^a^
WMS-GSH-0	1483 ± 4^d^	344 ± 5^e^	1139 ± 18^c^	482 ± 2^e^	138 ± 2^b^
WMS-GSH-5	1445 ± 2^e^	418 ± 7^d^	1027 ± 19^d^	538 ± 10^d^	120 ± 3^c^
WMS-GSH-10	2099 ± 7^c^	470 ± 1^c^	1629 ± 4^b^	566 ± 1^c^	96 ± 4^d^
WMS-GSH-15	2737 ± 7^b^	556 ± 14^b^	2181 ± 11^a^	673 ± 6^b^	117 ± 1^c^

LAS					
Native	326 ± 7^c^	258 ± 7^e^	68 ± 7^c^	291 ± 1^d^	33 ± 1^a^
LAS-GSH-0	647 ± 4^b^	502 ± 4^c^	145 ± 20^b^	522 ± 7^c^	20 ± 7^c^
LAS-GSH-5	636 ± 1^b^	484 ± 3^d^	152 ± 7^a^	535 ± 4^b^	21 ± 2^c^
LAS-GSH-10	666 ± 2^a^	520 ± 8^a^	146 ± 14^b^	548 ± 13^a^	28 ± 4^ab^
LAS-GSH-15	665 ± 2^a^	514 ± 2^bc^	151 ± 9^a^	538 ± 1^b^	24 ± 1^b^

HAS					
Native	14.00 ± 2.00^d^	14.00 ± 0.51^c^	8.00 ± 0.56^c^	16.00 ± 7.00^b^	2.00 ± 0.88^b^
HAS-GSH-0	34.00 ± 1.00^b^	19.00 ± 1.00^ab^	15.00 ± 0.82^b^	20.00 ± 1.00^a^	1.00 ± 0.54^c^
HAS-GSH-5	20.00 ± 1.00^cd^	19.00 ± 0.56^ab^	1.00 ± 0.21^d^	22.00 ± 1.00^a^	3.00 ± 0.53^ab^
HAS-GSH-10	16.00 ± 2.00^d^	16.00 ± 0.45^b^	6.00 ± 0.40^c^	20.00 ± 1.00^a^	4.00 ± 0.61^a^
HAS-GSH-15	39.00 ± 1.00^a^	21.00 ± 0.22^a^	18.00 ± 1.00^a^	20.00 ± 1.00^a^	1.00 ± 0.58^c^

Values are means ± SD in a column. Different letters represent a significant difference between data in the same amylose/amylopectin ratio (*p* < 0.05).

However, the values of LAS samples indicated that the pasting properties were independent from GSH and mainly affected by HHP-gelatinization. The PV and SB of HAS were slightly affected by the concentration of GSH. These results confirmed that GSH could interact with both amylose and amylopectin. Also, the GSH could be used as a formula material for maize starch with high amylose without shift the pasting properties in baking or other gelatinization process.

### 3.5 *In vitro* digestibility

As DG is closely related to *in vitro* digestibility, the *in vitro* α-amylase and glucosidase activities of WMS, LAS, and HAS with different DG values are shown in Table 3. Gelatinized starch showed a significant difference compared to native starch, except for WMS. Generally speaking, although the pattern of disruption was different, the disruption of the granules appeared progressively with the increase of DG. The results for WMS indicated that DG was the predominant factor influencing the RS contents, with trace amounts of RS possibly attributed to residual short-range molecular order in gelatinized samples (Liu et al., 2020). However, the RS contents of LAS were influenced by the GSH contents and showed a negative correlation with DG. The results indicated that RS might result from amylose/GSH or amylopectin/GSH crosslinking rather than the molecular orders in starch granules. Additionally, crosslinking enhanced the resistance ability of gelatinized LAS, especially with a GSH content of 10 %. Interestingly, the DG of HAS increased with the increase of GSH, while RS showed no significant difference. This result might be attributed to the addition of GSH inhibiting the formation of crystalline structure, with RS primarily related to the amorphous structure.

## 4 General discussion

**Fig. 6:**
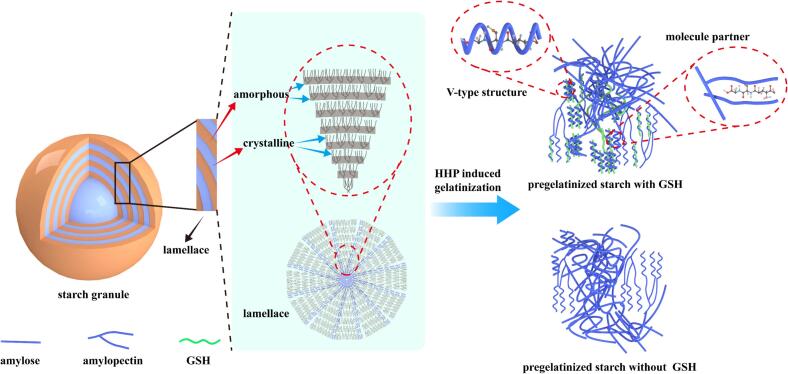
The interaction between GSH and maize starch.

As reflected by the DG values, the WMS was nearly completed gelatinized by HHP treatment, while as the amylose content increased the DG valuse decreased, which means the ratio of amylose/amylopectin defining the DG during the HHP treatments. However, it was interesting to find that as the increase of GSH in LAS and HAS, the DG value increase expect LAS-GSH-10. The results suggested that the GSH interacted with amylopectin elongated starches with amylopectin chain length (DP > 25), which prefer a B type starch (Klostermann, Buwalda, Leemhuis, de Vos, & Schols, 2021). In general, A-type starch was more resistant to digestive enzymes than B-type starch, the higher RS of LAS and HAS indicated the role of V type starch as reflected by the results of XRD. Thus, a remarkable improvement of RS would be expected for ratios of amylose/amylopectin between LAS and HAS, but it is not the case in this study. On the other hand, the increase of GSH in WMS and LAS was accompanied by an increase in B_2_ chains, especially at a GSH content of 10 %, suggested that at this content of GSH the crosslinking is complicate (Fig. 6). Also, the degradation of the A chains of HAS was obvious, and B_3_ chains slightly increased conformed the exist of V type starch formed by GSH and maize starch.

Further evidence for the formation of amylose-GSH/amylopectin-GSH complexes could be inferred from the FWHM values at 480 cm^−1^ and the peak ratio at 1045 cm^−1^/1022 cm^−1^ in IR analysis. The crosslinking of GSH-amylopectin led to the formation of short-range molecular order during HHP-gelatinization, whereas this effect was not observed in LAS and HAS, suggesting that GSH mainly interacts with amylose in LAS and HAS. However, the types of crystallites in LAS and HAS changed from A to C, possibly due to the lower DG values. Based on these findings, the presence of an amylose-GSH-amylopectin complex may explain the high RS contents in LAS samples, and the amylose-GSH complex could be more resistant to digestion than the amylopectin-GSH complex.

## 5 Conclusions

Pre-gelatinized starch with GSH was prepared *via* HHP with different amylose/amylopectin ratios. Morphological analysis using SEM revealed that GSH permeated into LAS and HAS samples without damaging the granules. Furthermore, the DG of LAS (59.36 %) and HAS (17.45 %) significantly increased with the addition of GSH. The GSH interacted with amylopectin and decreased the short-range ordered structure in WMS. Additionally, the type of crystallinity changed to C and V types in LAS and HAS, respectively, which further restricted starch retrogradation. Overall, these results indicated that GSH was involved in the rearrangement of starch crystallites based on amylose/amylopectin structures. However, a limited number of starch samples might also limit the generalizability of the findings to other types of starches or broader applications. Overall, this study provided valuable insights for the processing of pre-gelatinized starchy foods of maize with GSH.

## CRediT authorship contribution statement

**Wei Zhang:** Methodology, Conceptualization. **Danxia Shi:** Methodology. **Wenming Dong:** Investigation, Methodology. **Hong Li:** Funding acquisition. **Xiaohui Liu:** Resources, Funding acquisition.

## Declaration of competing interest

The authors declare that they have no known competing financial interests or personal relationships that could have appeared to influence the work reported in this paper.

## Data Availability

Data will be made available on request.
